# Construction of a High-Density Recombination Bin-Based Genetic Map Facilitates High-Resolution Mapping of a Major QTL Underlying Anthocyanin Pigmentation in Eggplant

**DOI:** 10.3390/ijms231810258

**Published:** 2022-09-06

**Authors:** Wenxiang Guan, Changjiao Ke, Weiqi Tang, Jialong Jiang, Jing Xia, Xiaofang Xie, Mei Yang, Chenfeng Duan, Weiren Wu, Yan Zheng

**Affiliations:** 1 Key Laboratory of Genetics, Breeding and Multiple Utilization of Crops, Ministry of Education, College of Agriculture/College of Life Sciences, Fujian Agriculture and Forestry University, Fuzhou 350002, China; 2 Fujian Provincial Key Laboratory of Crop Breeding by Design, Fujian Agriculture and Forestry University, Fuzhou 350002, China; 3 Marine and Agricultural Biotechnology Laboratory, Fuzhou Institute of Oceanography, Minjiang University, Fuzhou 350108, China

**Keywords:** eggplant, recombination bin-based genetic map, anthocyanin pigmentation, QTL mapping

## Abstract

High-density genetic maps can significantly improve the resolution of QTL mapping. We constructed a high-density recombination bin-based genetic map of eggplant based on 200 F_2_ plants from an interspecific cross (*Solanum melongena* × *S. incanum*) using the whole genome resequencing strategy. The map was 2022.8 cM long, covering near 99% of the eggplant genome. The map contained 3776 bins, with 3644 (96.5%) being effective (position non-redundant) ones, giving a nominal average distance of 0.54 cM and an effective average distance of 0.56 cM between adjacent bins, respectively. Using this map and 172 F_2:3_ lines, a major QTL with pleiotropic effects on two anthocyanin pigmentation-related traits, leaf vein color (LVC) and fruit pericarp color (FPC), was steadily detected in a bin interval of 2.28 cM (or 1.68 Mb) on chromosome E10 in two cropping seasons, explaining ~65% and 55% of the phenotypic variation in LVC and FPC, respectively. Genome-wide association analysis in this population validated the QTL and demonstrated the correctness of mapping two bins of chromosome E02 onto E10. Bioinformatics analysis suggested that a WDR protein gene inside the bin interval with reliable effective variation between the two parents could be a possible candidate gene of the QTL.

## 1. Introduction

During the development of quantitative genetics, one of the most important events was the advent of molecular marker technologies, which enables the construction of genetic maps for mapping quantitative trait loci (QTLs), dissecting the genetic basis of a quantitative trait into individual Mendelian factors. The resolution of QTL mapping is determined by many factors, such as the complexity and heritability of the trait, the type and size of the population, the marker density of the genetic map, and the power of the statistical method. High marker density is a precondition for high mapping resolution. The fast development of the next-generation sequencing (NGS) technologies has dramatically reduced the costs of high-throughput genotyping and made it practical to construct a high-density genetic map. 

There can be two strategies of NGS-based genotyping for constructing a high-density genetic map. One is genotyping by partial genome sequencing (PGS), namely, genotyping individuals by sequencing a selected part of the genome. A few methods for PGS have been developed, such as restriction site-associated DNA sequencing (RAD-seq) [1,2], genotyping-by-sequencing (GBS) [3], and specific length amplified fragment sequencing (SLAF-seq) [4]. The merit of PGS is that it does not need a reference genome sequence and can reduce the cost, especially useful for the species with large genome size. So far, high-density genetic map construction has been mainly performed based on this strategy. It has been used in many plant species, such as soybean [5], cucumber [6], chickpea [7], durum wheat [8], rice [9,10], grape [11], *Brassica rapa* [12], wheat [13], and potato [14].

The other strategy is genotyping by whole genome resequencing (WGR). This strategy was first proposed by Huang et al. [15]. It roughly involves four steps: (1) resequencing the whole genome of each individual in the mapping population at a very low sequencing depth; (2) collectively examining genome-wide polymorphisms for genotype calling and recombination breakpoint determination using a sliding window approach; (3) dividing the genome into recombination bins; and (4) analyzing the genetic linkage among the bins. As WGR can identify all polymorphisms in a population in principle, it has the potential to uncover all recombination events in the population so as to maximize the density and the resolution of the genetic map. This is an advantage of WGR over PGS. Apart from in rice [15,16], this strategy has been used in some other species, such as soybean [17], maize [18], pepper [19], melon [20], peanut [21], and radish [22]. As reference genome sequence is available in more and more species and NGS becomes cheaper and cheaper, the WGR strategy will be used more widely in high-density genetic map construction.

Eggplant (*Solanum melongena* L.) is an important vegetable crop cultivated and consumed worldwide. To facilitate the genetic studies in eggplant, a number of genetic maps of eggplant have been constructed since the early of this century [23,24,25,26,27,28,29,30,31,32]. Most of the maps, however, were constructed based on traditional molecular markers and had a low marker density. Recently, two high-density genetic maps were constructed with the PGS strategy in eggplant. One was constructed based on an interspecific (*S. melongena* × *S. linnaeanum*) F_2_ population consisting of 121 individuals using the SLAF-seq approach [33]. The map comprises 2122 single nucleotide polymorphism (SNP) markers, with a total length of 1530.75 cM and an average distance of 0.72 cM between adjacent markers. The other map was constructed based on a population of 163 recombinant inbred lines (RILs) derived from a cross between two cultivated eggplant varieties using the GBS approach [34]. The map contains 7249 SNP markers, with a total length of 2169.23 cM and an average distance of 0.4 cM between adjacent markers. More recently, a bin-based genetic map (or termed bin map for short) was constructed based on a population of 100 F_2_ individuals developed from an interspecific cross (*S. melongena* × *S. insanum*) using the WGR strategy [35]. The map consists of 3918 bins, with a length of 1384.62 cM in total and a distance of 0.58 cM between adjacent bins on average. These maps have exhibited the merit of high marker density for increasing the resolution of gene/QTL mapping. 

Eggplant is rich in anthocyanins, which are beneficial for human health [36]. So, increasing anthocyanin content is an important goal in eggplant breeding. For this purpose, it is necessary to understand the genetic basis and regulatory mechanism of anthocyanin accumulation in eggplant. In the past decade, especially in recent years, remarkable progress has been made in the molecular genetic research of anthocyanin accumulation in eggplant. A number of QTLs related to anthocyanin accumulation have been mapped on all eggplant chromosomes except chromosome E04 [23,34,37,38], and some anthocyanin metabolism-related genes have been identified using the approaches of reversed genetics, including transcriptomic or proteomic analysis [39,40,41] and homologous or candidate gene analysis [42,43,44,45]. However, as many genes/QTLs are involved in the control of anthocyanin accumulation, the genetic basis and regulatory mechanism underlying the anthocyanin metabolism in eggplant is still far from being clarified.

In this study, we constructed a high-density recombination bin-based genetic map based on an inter-specific F_2_ population and applied it to mapping QTLs for anthocyanin pigmentation in eggplant. We identified a major QTL displaying pleiotropic effects on the anthocyanin pigmentation in both leaf and fruit and demonstrated that the high-density map facilitated high-resolution mapping of the major QTL and prediction of its possible candidate gene. 

## 2. Results

### 2.1. Genotyping Results

After sequencing, ~178.06 M and 138.30 M clean reads were obtained in the parental lines YZQ (*S. melongena*) and YQ (*S. incanum*), respectively, of which 98.69% and 98.83% could be mapped to the reference genome. The number of clean reads obtained in each of the 200 F_2_ plants was ~17.83M on average, varying 13.51 M–31.33 M, of which 99.12% (97.48–99.51%) could be mapped to the reference genome. The Q20 of each F_2_ plant varied 94.72–97.75%, with an average of 96.75%. These results indicated that the sequencing data were good in quality overall.

**Figure 1:**
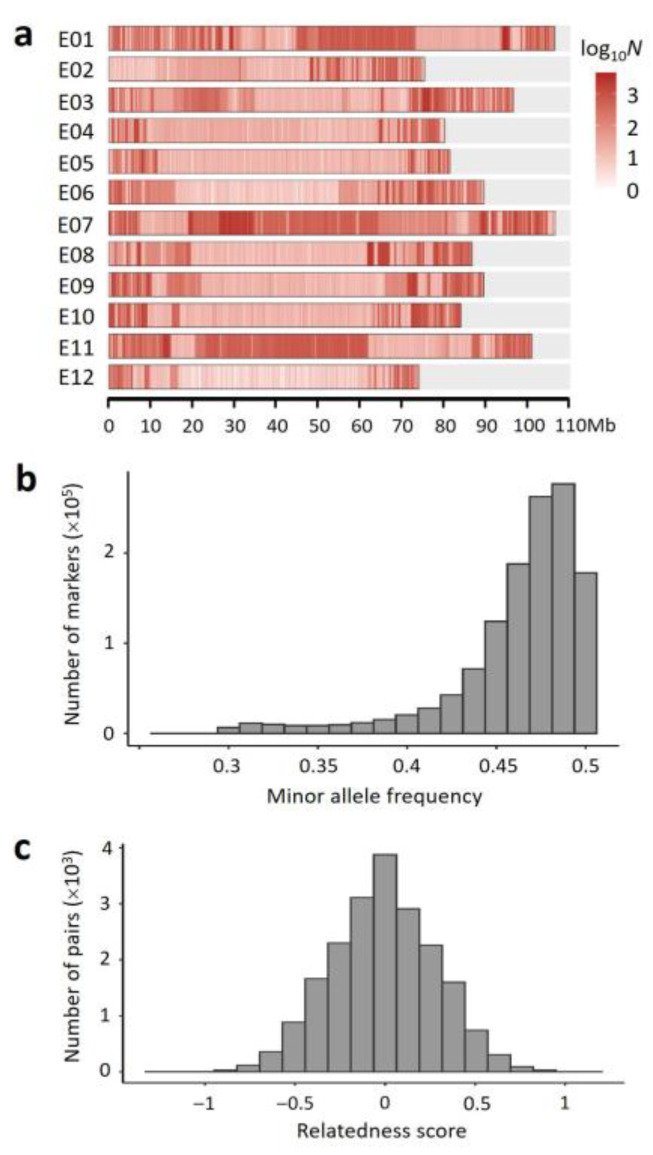
Characteristics of the markers and the F_2_ population: (**a**) distribution of marker density (number of markers per Mb) in each chromosome. The color scale is on the right; (**b**) distribution of minor allele frequency (MAF) of the markers in the F_2_ population; and (**c**) distribution of relatedness score between F_2_ individuals.

Based on the sequencing data, a set of 1,273,701 markers (SNPs and short InDels) meeting the three criteria (diallelic; homozygous in YZQ; minor allele frequency, MAF ≥ 0.3) were selected in the F_2_ population. The markers were not evenly distributed in the genome (Figure 1a). In most of the chromosomes, markers were mainly located close to the two ends. In chromosomes E01, E07 and E11, however, there was a long segment with higher marker density in the middle region, respectively. In addition, marker density was very low in the left terminal region of E02. The proportion of missing marker genotype data in each F_2_ plant was ~0.14 on average, varying 0.03–0.26. So, the number of informative markers in each F_2_ plant would not be smaller than 1.27 M × (1 − 0.26) = 0.94 M, which should be adequate for constructing a recombination bin-based genetic map.

To evaluate the quality of the F_2_ population, we analyzed the following parameters based on the marker data: (1) The MAFs of markers. The MAFs were largely distributed between 0.45 and 0.5 in the F_2_ population, peaking at ~0.4875 (Figure 1b), very close to the expected peak value 0.5. (2) The Proportion of Heterozygous marker Genotypes (PHG) in each F_2_ plant. The observed PHG ($p_{o}$) was ~0.25 on average, varying 0.10–0.42. Theoretically, the expected PHG ($p_{e}$) in an F_2_ individual is 0.5. However, because there is a probability ($p_{m}$) that a heterozygous genotype is misjudged as a homozygous genotype depending on the sequencing depth, $p_{o}$ must be smaller than $p_{e}$. It is easy to find that $p_{m}=1/2^{k-1}$, where *k* is sequencing depth. In this study, the sequencing depth for each F_2_ plant was ~2×. Therefore, $p_{m}\approx 0.5$ on average. It can be predicted that the average $p_{o}=p_{e}\times \left(1-p_{m}\right)=0.5\times \left(1-0.5\right)=0.25$. This is consistent with the average PHG observed. (3) The genetic relationship between individuals [46]. The standardized relationship coefficient (or termed relatedness score) approximately followed a normal distribution in the F_2_ population (Figure 1c), suggesting that the genetic segregation in the F_2_ population was symmetric and unbiased overall. The above results together indicated that the F_2_ population was normal in structure, suitable for genetic mapping.

### 2.2. Recombination Bin-Based Genetic Map Constructed

**Figure 2:**
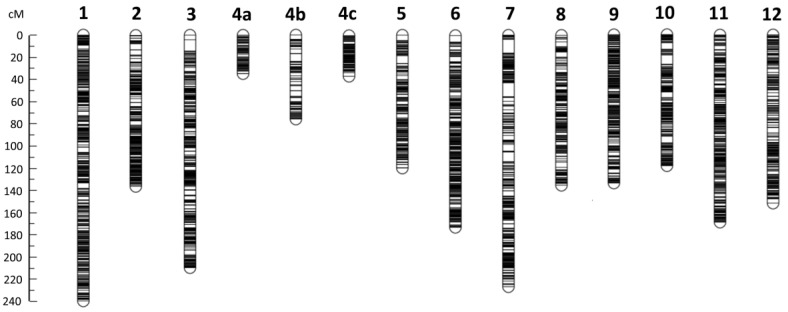
High-density recombination bin-based genetic map constructed in eggplant. The position of each bin is indicated by a horizontal line. The genetic distance scale in cM is shown on the left. Chromosome E04 was divided into three linkage groups (4a, 4b and 4c).

The genetic map constructed based on the F_2_ population was 2022.8 cM in length, containing 14 linkage groups and 3776 bins (Table 1; Figure 2; Table S1). The bin length was 135.9 kb on average, varying from 10 to 16,490 kb, with ~82%, 87%, 90%, 94% and 96% smaller than or equal to 150, 200, 250, 300 and 400 kb, respectively. The total length of the bins was ~498.32 Mb, accounting for 46.44% of the eggplant genome sequence, indicating that about half of the genome sequence was not included as bins in the genetic map. There were 132 redundant bins in terms of the position in the genetic map (when there were multiple bins at a position in the map, they function only as one effective marker, so the extra bins were redundant). So, the number of effective bins (bin markers) in the map was 3776 − 132 = 3644. Each chromosome corresponded to a linkage group in the map except for E04, which was divided into three linkage groups (LG4a, 4b and 4c). Overall, the bin density was high throughout the map. The nominal average distance (between adjacent bins, including redundant bins) in each linkage group varied from 0.42 cM (LG4c) to 0.78 cM (LG4b), and was 0.54 cM in the whole map; whereas the effective average distance (redundant bins excluded) was close to the nominal one in each linkage group, varied from 0.42 cM (LG4c) to 0.98 cM (LG4b), and was 0.56 cM in the whole map (Table 1). The largest spacing between adjacent bins was 12.3 cM in LG7 (Figure 2). The physical span (from the beginning of the first bin to the end of the last bin) of each linkage group (or the sum of the three linkage groups of E04) was exactly or nearly equal to the full length of the corresponding chromosome except for LG2 (Table 1). In total, the genetic map physically spanned ~1.06 Gb, covering near 99% of the eggplant genome (Table 1). Therefore, the genetic map basically represented the whole eggplant genome.

The genetic map constructed based on the F_2_ population was 2022.8 cM in length, containing 14 linkage groups and 3776 bins (Table 1; Figure 2; Table S1). The bin length was 135.9 kb on average, varying from 10 to 16,490 kb, with ~82%, 87%, 90%, 94% and 96% smaller than or equal to 150, 200, 250, 300 and 400 kb, respectively. The total length of the bins was ~498.32 Mb, accounting for 46.44% of the eggplant genome sequence, indicating that about half of the genome sequence was not included as bins in the genetic map. There were 132 redundant bins in terms of the position in the genetic map (when there were multiple bins at a position in the map, they function only as one effective marker, so the extra bins were redundant). So, the number of effective bins (bin markers) in the map was 3776 − 132 = 3644. Each chromosome corresponded to a linkage group in the map except for E04, which was divided into three linkage groups (LG4a, 4b and 4c). Overall, the bin density was high throughout the map. The nominal average distance (between adjacent bins, including redundant bins) in each linkage group varied from 0.42 cM (LG4c) to 0.78 cM (LG4b), and was 0.54 cM in the whole map; whereas the effective average distance (redundant bins excluded) was close to the nominal one in each linkage group, varied from 0.42 cM (LG4c) to 0.98 cM (LG4b), and was 0.56 cM in the whole map (Table 1). The largest spacing between adjacent bins was 12.3 cM in LG7 (Figure 2). The physical span (from the beginning of the first bin to the end of the last bin) of each linkage group (or the sum of the three linkage groups of E04) was exactly or nearly equal to the full length of the corresponding chromosome except for LG2 (Table 1). In total, the genetic map physically spanned ~1.06 Gb, covering near 99% of the eggplant genome (Table 1). Therefore, the genetic map basically represented the whole eggplant genome.

According to the reference genome sequence, the order of the three linkage groups of E04 is LG4a-LG4b-LG4c (Table S1). However, the three linkage groups could not be linked accordingly because the estimated genetic distance between LG4a and LG4b and that between LG4b and LG4c were both very large (>90 cM). When there was no restriction on the order, LG4b still could not be linked to LG4a and LG4c. Hence, LG4b appeared to be genetically independent from LG4a and LG4c. Surprisingly, LG4a and LG4c could be linked in the absence of LG4b with a seemingly acceptable gap (25.8 cM) between them.

There were 45 bins that could not be mapped according to the physical order in the reference genome, but 13 of them were successfully mapped to the linkage groups of other chromosomes, including one from E12 to LG5; two from E02 to LG10; one from E04 and three from E05 to LG9; and one from E02, E05 and E09 each and three from E10 to LG11 (Table S2). The two bins of E02 (B02_129 and 130) mapped to LG10 were adjacent and therefore actually represented a continuous sequence in the reference genome. Similar situations also existed for the three bins of E05 (B05_207, 208 and 209) mapped to LG9 and the three bins of E10 (B10_194, 195 and 196) mapped to LG11 (Table S2). After mapped to LG10 and LG11, the two bins of E02 and the three bins of E10 were merged (as B02_129 and B10_194), respectively (Table S1).

**Figure 3:**
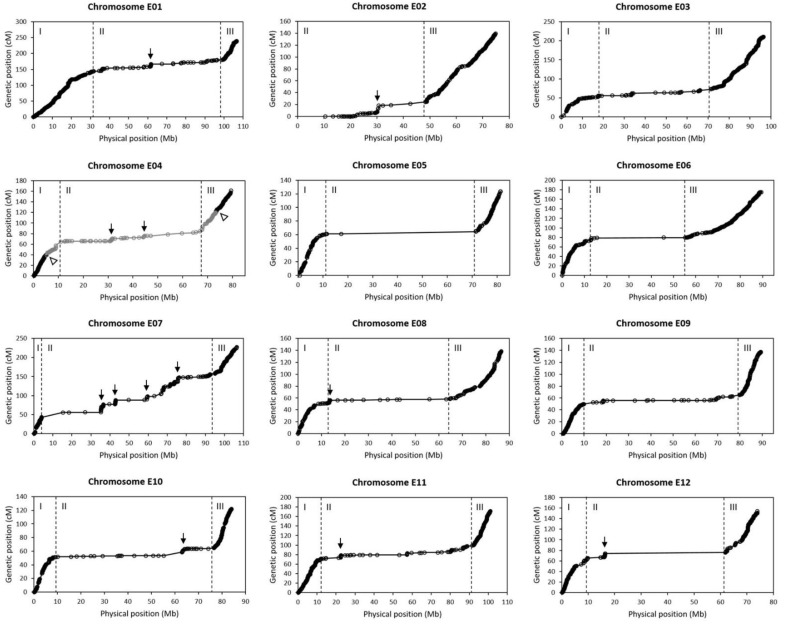
Relationship of the genetic positions with the physical positions of the bins in each chromosome. Each circle indicates a bin. The physical position of a bin is represented by the center of the bin. The vertical dashed lines divide a chromosome into three regions. Arrows indicate the possible hotspots of genetic recombination in region II. For chromosome E04, the middle gray part is LG4b, while the left and right black parts are LG4a and LG4c, respectively. The blank arrowheads indicate the breakpoints between linkage groups.

By comparing the genetic map with the physical map (reference genome), it was found that each chromosome could be divided into three regions (I, II and III) with two obvious distinguishing features (Figure 3). One feature is bin density. In all the chromosomes except for E02, regions I and III had a very high bin density, while region II had much lower bin density (Figure 3). This was basically consistent with the marker density distribution in the genome (Figure 1a). In addition, this result also indicated that the sequences not contained as bins in the genetic map were mainly in the middle region (region II) of each chromosome. The other feature is Ratio of Genetic distance to Physical distance (RGP). The average RGPs (=total genetic length/total physical length) in regions I and III were high in general, while that in region II was very low (Table 2). This can be intuitively seen in Figure 3, where the overall slope of the genetic position-physical position curve appears to be very small in region II in each chromosome, suggesting that a very large physical distance is only equivalent to a small genetic distance in region II. Therefore, genetic recombination must occur mainly in regions I and III, but rare in region II. This explains why a high-density genetic map spanning the whole chromosome could be constructed although there were very few bins from region II.

Among the 12 chromosomes, three appeared to be special. The first was E02. Its first 10 Mb region was not covered by the genetic map (Figure 3). The reason was that the marker density in this region was very low (Figure 1a). It was unable to identify the region I in E02 (Figure 3). The marker density distribution also suggested that there was no region I in E02 (Figure 1a). Hence, E02 only contained two regions: region II with low average RGP and region III with high average RGP (Table 2). The second was E04. Its regions I and III were both broken into two parts. LG4b spanned the three regions, whereas LG4a and LG4c were only a part of region I and region III, respectively (Figure 3). The third was E07. The average RGP in the region II of E07 was much larger than those of other chromosomes (Table 2; Figure 3). In addition, although region II had a small average RGP in general, it contained some positions displaying obviously steep slopes (large RGPs) in some chromosomes, especially in E02, E04 and E07 (Figure 3). These positions must be the hotspots of genetic recombination.

### 2.3. QTLs Mapped and Candidate Gene Predicted

**Figure 4:**
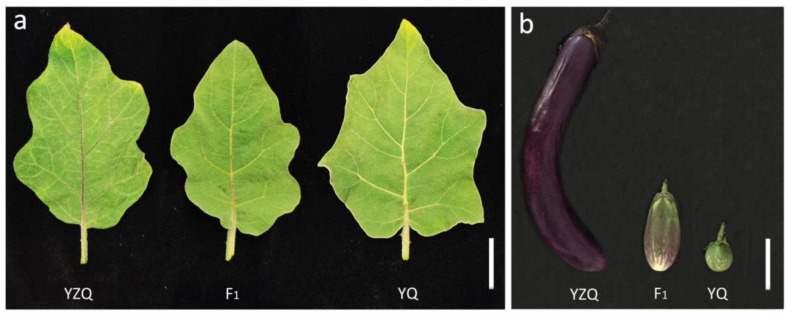
Leaves (**a**) and fruits (**b**) of YZQ, F_1_ and YQ. Bar = 3 cm in (**a**) and 5 cm in (**b**).

Based on the field experiments of 172 F_2:3_ lines in two different cropping seasons, a major QTL was mapped on E10 in each of the two traits, leaf vein color (LVC) and fruit pericarp color (FPC), in both seasons, named as *qLVC10* and *qFPC10b*, respectively (Table 3; Figure S1). The positions and bin intervals of the major QTLs detected in different traits and different seasons were completely the same, all being 63 cM and B10_240-B10_249 (60.97–63.25 cM), respectively (Table 3; Table S1). The results suggested that these major QTLs were actually the same QTL with pleiotropic effects, and the mapping result was reliable and of high resolution (within a marker interval of 2.28 cM) for a primary mapping population of a median size. The major QTL had very high heritability, which could explain ~65% and 55% of the phenotypic variation in LVC and FPC, respectively (Table 3). It showed very large additive effects but negligible dominance effects in the two traits (Table 3). The allele from the cultivated parent YZQ increased the trait value, while the allele from the wild parent YQ acted negatively (Table 3). This was consistent with the phenotypes of the two parents, namely, the colors of leaf vein and fruit pericarp were purple in YZQ, but not purple (no anthocyanin pigmentation) in YQ, while those in F_1_ were medium between the two parents (Figure 4).

Apart from the major QTL, there were three and two minor QTLs detected in LVC and FPC, respectively (Table 3). All of the minor QTLs were detected only in the first season, and there were no common minor QTLs between the two traits. In addition, the LOD scores of all the minor QTLs except *qFPC6* were only slightly over the threshold. Therefore, most of these minor QTLs detected might not be very reliable.

Based on the marker (SNP and short InDel) genotype data of the F_2_ population, we also performed genome-wide association study (GWAS) on the two anthocyanin pigmentation traits using the method of mixed linear model (MLM) implemented by the R program rMVP [47]. The GWAS results also indicated the existence of a major QTL in the region between 60 Mb and 75 Mb on chromosome E10 (Figure S2). In addition, some of the minor QTLs mapped by the linkage analysis (e.g., *qFPC6*) also showed obvious signal in the GWAS (Figure S2; Table 3). Therefore, the results of linkage analysis and GWAS were consistent. However, it is noticeable that there was an obvious steady –log10(P) peak detected at ~30 Mb on chromosome E02 in both traits and both cropping seasons (Figure S2), but no QTL mapped at that position (Figure S1). By careful examination, we found that this –log_10_(P) peak was just located at the two bins, B02_129 and B02_130, which were mapped to E10 in the genetic map, located inside the bin interval of the major QTL and completely linked with B10_249 (Tables S1 and S2; Table 3). This explains the cause of the –log_10_(P) peak on E02.

The bin interval of the major QTL (B10_240-B10_249) was 1.68 Mb wide in the reference genome (Table 3), harboring 103 genes according to the annotation (Table S3). In addition, it is interesting that the bin B02_129 from E02 happened to be mapped between B10_240 and B10_249, completely linked with (0 cM to) B10_249 (Table S1). The size of B02_129 was 400 kb, in which only four genes were annotated (Table S3). Among these annotated genes, only one (*Smechr1001624*) met the criteria for reliable effective variation between the wild parent YQ and the cultivated parent YZQ. A SNP (A/G) between the two parents was found at 64,566,331 bp inside *Smechr1001624*, making the codon AGT in YQ altered into GGT in YZQ and therefore leading to a change of the 743rd amino acid in the encoded protein from Serine in YQ into Glycine in YZQ. *Smechr1001624* is predicted to encode a WD repeat-containing (WDR) protein (Table S3). WDR proteins are a very large family. It has been found that WDR proteins play a critical role in the regulation of anthocyanin biosynthesis in many plant species [40,48,49,50,51,52]. WDR physically interacts with MYB and bHLH to form a transcriptional complex, which participates the regulation of the flavonoid pigment pathway [50]. The above information suggests that *Smechr1001624* could possibly be a candidate gene for the major QTL.

## 3. Discussion

In this study, we constructed a high-density recombination bin-based genetic map of eggplant using an interspecific (*S. melongena* × *S. incanum*) F_2_ population consisting of 200 individuals (Table 1). Only one recombination bin-based genetic map of eggplant has been reported before [35] (named as Qian’s map here). Qian’s map is also constructed based on an interspecific F_2_ population by means of low-depth resequencing (~1.4× for each individual on average), but the wild parent is a different species (*S. insanum*), and the population is smaller (only 100 individuals). Qian’s map is 1384.6 cM in length (~32% shorter than ours) but contains 3918 bins (~4% more than those in our map). However, most (2370/3918 = 60.5%) of the bins in Qian’s map are redundant; only 1548 are effective bins (~42% of the number in our map). Therefore, the effective average distance between adjacent bins in Qian’s map is ~0.90 cM, which is ~60% larger than that in our map. So, our map is much longer and much denser than Qian’s map. Since the most apparent difference between our study and that of Qian et al. is that the population we used is twice as large as that used by them, population size must be the major cause leading to the significant difference between our map and Qian’s map in total length and effective density, indicating the importance of population size for high-density genetic map construction. Theoretically, the number of recombinations occurring in a population is directly proportional to the population size, while the number of markers (mainly SNPs) revealed by sequencing in a population is usually large enough to identify every genetic recombination occurred. Therefore, population size is the key for achieving the high density of a genetic map. In principle, the population size should be as larger as possible as long as the budget allows.

The method of constructing recombination bin-based genetic map by low-depth resequencing was originally proposed for permanent (e.g., RIL) populations [15]. So, most of the bin maps reported were constructed using permanent populations [15,16,17,18,19,21]. Nevertheless, F_2_ populations have also been used for constructing bin maps [20,22,35], although it suffers a problem that the heterozygous genotypes of markers are often miscalled as homozygous genotypes under low-depth sequencing. A way to deal with this problem is to predict the most possible marker genotypes by integrating the information of multiple nearby markers. Following this principle, the method of Huang et al. [15] was still adopted for F_2_ populations after modification in those studies reported. In the present study, we developed a different method for the bin map construction based on F_2_ populations. The results demonstrated the feasibility of our method. Although the current approaches appear to be effective, the high proportion of misjudgment on heterozygous genotypes due to low sequencing depth may still bring about genotyping mistakes, especially impeding precise identification of the exchange breakpoints of chromosomes, so as to affect the quality of the map. It has mentioned in Section 2.1 that the probability of a heterozygous genotype being misjudged as a homozygous genotype is $p_{m}=1/2^{k-1}$, where *k* is sequencing depth. According to this formula, it can be found that the probability would be <1/16 when sequencing depth > 5×. So, a little higher (than low) sequencing depth is beneficial to the bin map construction based on F_2_ populations. For example, Luo et al. constructed a bin map in radish using an average sequencing depth of 7.2× for each F_2_ individual [22]. However, increasing sequencing depth will increase the cost. 

Our study revealed that the relationship of genetic position with physical position in each eggplant chromosome usually appeared as a curve of three slopes, namely, steep slope → gentle slope → steep slope; but in chromosome E02 the curve only contained two slopes, namely, gentle slope → steep slope (Figure 3). Similar phenomenon was observed in the study of Qian et al. [35]. In particular, E02 also displays a curve of two slopes in their studies, suggesting that the exceptive performance of E02 is not an accidental result but a characteristic of E02. The nonlinear relationship between genetic position and physical position suggests that it is inappropriate to use genome-wise average RGP but region-wise average RGP in the studies of map-based cloning of genes. 

Chromosome E04 was split into three linkage groups in the bin map constructed in this study. Physically, the three linkage groups were continuous according to the reference genome (Table S1). Each linkage group looked normal and they together could form a continuous three-slope relationship curve between genetic position and physical position similar to those of other chromosomes (Figure 3). So, the breakage of E04 as three linkage groups did not seem to be caused by experimental errors or data analysis errors. The result was really puzzling. Perhaps, the breakpoints between LG4a and LG4b and between LG4b and LG4c were two recombination hotspots with very high recombination rates in this population. Anyway, the separated linkage groups of E04 may not greatly affect the application of the map to QTL mapping.

In this study, we mapped a major QTL for anthocyanin pigmentation on E10. A major QTL for anthocyanin pigmentation has also been mapped on E10 before [23,34,37], which is estimated to be located at about 94-95 Mb in E10 [34] in the reference genome of *S. melongena* 67/3 line [53]. According to alignment analysis, we found that this location approximately corresponded to 66-67 Mb in the reference genome of SME-HQ1315 [54], very close to location of the major QTL mapped in this study (Table 2). So, we suspect that the reported major QTL and the one mapped in this study might be the same.

We have seen in the results that the GWAS validated the existence of the major QTL on chromosome E10. Besides, it also proved the correctness of the result that the two bins of chromosome E02 (B02_129 and B02_130) according to the reference genome were mapped to E10 in this study. This demonstrated an additional usefulness of high-density bin-based genetic maps for correcting the mistakes of genome sequence assembly and discovering the false localization of a major QTL by GWAS due to the incorrect genome sequence assembly. 

## 4. Materials and Methods

### 4.1. Plant Materials

An eggplant (*S. melongena*) cultivar Yanzhiqie (YZQ; accession ID: V06B0149) and a wild relative (*S. incanum*) germplasm Yeqie (YQ; accession ID: V06B0864) kindly provided by the Institute of Vegetables and Flowers, Chinese Academy of Agricultural Sciences were used as parents. The two parents are distinct in many traits including anthocyanin pigmentation. YZQ shows purple color in leaf vein and fruit pericarp, while YQ does not (Figure 4). A YZQ plant (female parent) and a YQ plant (male parent) were crossed, and an F_1_ plant was selfed to produce an F_2_ population of 200 plants. Further, an F_2:3_ population of 172 lines were developed by selfing the F_2_ plants.

### 4.2. Detection of Marker Genotypes by Whole Genome Resequencing

Genomic DNA was extracted from young leaves of every F_2_ plant and the two parents. The DNA samples were sequenced based on pair-end libraries using either the BGISeq500 platform (for the F_2_ plants, conducted by BGI) or the Illumina NovaSeq platform (for the parents, conducted by Novagene). The sequencing depth for each F_2_ plant was ~2×, while those for parents YZQ and YQ were ~53× and 42×, respectively. Adapter removing and quality control of reads were performed using the software Fastp [55] (https://github.com/OpenGene/fastp, Chen S., Shenzhen, China). Clean reads (150 bp in length each) were mapped to the eggplant reference genome SME-HQ1315 [54] (http://eggplant-hq.cn, accessed on 10 June 2020) using the software BWA-MEM [56] (http://maq.sourceforge.net, Li H., Cambridge, UK) and then sorted using the software SAMtools [57] (http://samtools.sourceforge.net, Li H., Cambridge, UK). Variants (including SNPs and short InDels) and their genotypes in the parents and the F_2_ plants were identified using the software FreeBayes [58] (v2, https://github.com/freebayes/freebayes, Garrison E., Boston, MA, USA), and the variants meeting the following criteria were selected as molecular markers: (1) diallelic; (2) homozygous in the parent YZQ; and (3) the minor allele frequency (MAF) ≥ 0.3 in the F_2_ population. According to the marker genotype information of the two parents, the genotype of every marker in each F_2_ plant was determined, denoted as 0 (for YZQ genotype), 1 (for heterozygous genotype) or 2 (for YQ genotype). 

### 4.3. Construction of Recombination Bin-Based Genetic Map

A main problem of using F_2_ population to construct recombination bin-based genetic map is that each marker has heterozygous genotype, which is easy to be misidentified as parental genotypes under low sequencing depth. For this reason, we designed a method different from that of Huang et al. [15] to determine recombination bins. We used a sliding window with fixed size (300 kb) to calculate a genotype index for judging the genotype of the window center in each F_2_ plant. The genotype index was defined as: $\mathrm{GI}=0\times p_{0}+1\times p_{1}+2\times p_{2}$, where $p_{0}$, $p_{1}$ and $p_{2}$ were the proportions of markers with the YZQ genotype, the heterozygous genotype and the YQ genotype within the window, respectively. Obviously, GI varied between 0 and 2. It would be close to 0 when the window was in a YZQ-genotype region, but close to 2 when in a YQ-genotype region. In a heterozygous region, as a heterozygous marker had an equal probability to be misidentified as either the YZQ genotype or the YQ genotype, the genotyping errors of different markers could be partially eliminated after averaging in the sliding window, making the GI value vary around 1. So, in principle, genomic positions with different genotypes could be distinguished according to their GI values. In order to use GI to discriminate genotype explicitly, we established the following function after trial to transform GI into integer: let GI = 0 (YZQ genotype) when GI ≤ 0.2; 1 (heterozygous genotype) when 0.2 < GI ≤ 1.8; and 2 (YQ genotype) when GI > 1.8. To get reliable or valid GI estimates, a minimum number of 10 markers was required in a window. The window moved with a step length of 10 kb from one end to the other on each chromosome. Thus, a GI estimate was obtained every 10 kb in the genome in each F_2_ plant.

Based on the integer GI data, co-segregations of adjacent positions in the F_2_ population were examined. A genomic segment containing a string of consecutive positions that completely co-segregated was defined as a recombination bin. So, every chromosome was partitioned into many bins. Each bin behaved as a separate unit for genetic recombination like a marker in the population. Using chi-square test, bins showing very serious deviation from the theoretical segregation ratio (1:2:1) in the F_2_ population (chi-square > 25, *p*-value < 105) were filtered out. Then, by fixing the order of the bins in each chromosome according to the reference genome sequence, the genetic distances between adjacent bins were calculated using the software QTL IciMapping v4.2 [59] (https://isbreedingen.caas.cn/software/qtllcimapping/294607.htm, accessed on 24 July 2019). For those bins that could not be mapped according to the expected order, each was taken as an inquiry bin to search for the target bin that was closely linked to it, using the option Rippling provided in the software. If a target bin was found, the inquiry bin was tried to be inserted into the map beside the target bin. After no more bins could be added into the map, the following procedure was conducted on each linkage group: (1) bin genotype errors detected by the linkage analysis were corrected according to the flanking bins; (2) bins that had the same genetic position and were physically consecutive were merged as a larger bin; and (3) linkage analysis was performed again. This procedure was repeated until no bin genotype errors could be found and no bins could be merged in the map. 

### 4.4. Field Experiment and Phenotyping

Field experiments of the F_2:3_ population with the two parents as controls were carried out twice in the farm of Fujian Agriculture and Forestry University at Yangzhong, Youxi (E118.485841, N26.287161) in two different cropping seasons in 2020. In the first season, seeds were sown in growing trays after pregermination on 20 February, and seedlings were transplanted into the field on 5 April. In the second season, seeds were sown on 9 May and seedlings were transplanted on 12 July. In both seasons, the seedlings to be transplanted were all at the age of five full leaves plus one emerging leaf. The seedlings were planted in a regime of 80 cm between rows and 100 cm between plants, under a randomized complete block design with two replicates. Five seedlings were planted for each F_2:3_ line in each replicate.

For each plant, leaf vein color (LVC) was observed from the adaxial side of a mature leaf at the stage when the second inflorescence on the main stem began to flower, measured as 0 (green), 1 (light purple), or 2 (purple); fruit pericarp color (FPC) was investigated from the fruits produced by the second inflorescence on the main stem at the stage of horticultural maturity, measured as 0 (white), 1 (greenish white), 2 (light green), 3 (green), 4 (dark green), 5 (purple), 6 (brown), or 7 (dark brown).

### 4.5. QTL Analysis and Candidate Gene Prediction

Mapping of QTLs underlying the color traits in the F_2:3_ population was performed based on the bin genotype data and the genetic map using the software QTL IciMapping v4.2 [59]. The LOD threshold at the overall (genome-wise) significance level of 0.05 was estimated by permutation test with 1000 replicates. Genome-wide association study (GWAS) was conducted to verify the mapped QTLs using the marker (SNP and short InDel) data and the method of mixed linear model (MLM) implemented by the R program rMVP [47]. Using the genome sequencing data of the two parents, possible candidate genes of a major QTL were predicted by examining reliable and effective coding sequence variations between the two parents within the QTL’s bin interval based on the following criteria: (1) the polymorphic site was homozygous with sufficient sequencing depth (counts ≥ 6) in both parents; (2) the variation could cause protein change between the two parents; and (3) the genotype of the polymorphic site in YZQ (or YQ) was the same as (or different from) that in the reference genome of HQ-1315, which is similar to YZQ in anthocyanin pigmentation [54].

## 5. Conclusions

In this study, a high-density recombination bin-based genetic map of eggplant was constructed based on an F_2_ population derived from an interspecific cross (*S. melongena* × *S. incanum*) using the whole genome resequencing strategy and was then applied to QTL analysis for anthocyanin pigmentation in leaf vein and fruit pericarp. A major QTL with pleiotropic effects on the two traits was steadily mapped in a small region on chromosome E10 in two cropping seasons, and a possible candidate gene of the QTL was identified. In addition, some mistakes of genome sequence assembly in the reference genome were found and corrected in the genetic map construction. Our results demonstrated the merit of high-density genetic map for high-resolution QTL mapping as well as for discovering and correcting the mistakes in genome sequence assembly.

## Figures and Tables

**Table 1:** Information of the recombination bin-based genetic map constructed in eggplant.

LG ^a^	Length (cM)	No. of Bins ^b^	Spacing (cM) ^c^	Span (Mb) ^d^	Full (Mb) ^e^	% ^f^
1	239.4	484 (474)	0.50 (0.51)	106.63	106.64	99.99
2	139.1	298 (282)	0.47 (0.50)	64.66	75.42	85.73
3	210.3	375 (364)	0.56 (0.58)	96.71	96.71	100.00
4a+4b+bc	160.8	281 (259)	0.58 (0.62)	79.16	80.28	98.60
4a	39.2	79 (78)	0.50 (0.51)	5.08	5.33	95.31
4b	80.4	104 (83)	0.78 (0.98)	67.92	67.92	100.00
4c	41.2	98 (98)	0.42 (0.42)	6.16	7.03	87.62
5	123.5	212 (209)	0.59 (0.59)	80.73	81.59	98.95
6	174.9	368 (358)	0.48 (0.49)	89.37	89.68	99.65
7	226.9	338 (334)	0.67 (0.68)	106.41	106.79	99.64
8	138.4	249 (241)	0.56 (0.58)	86.62	86.83	99.76
9	136.9	314 (298)	0.44 (0.46)	89.14	89.64	99.44
10	121.7	244 (224)	0.50 (0.55)	84.17	84.17	100.00
11	170.9	348 (339)	0.49 (0.51)	101.22	101.22	100.00
12	154.3	265 (262)	0.58 (0.59)	73.98	74.17	99.74
Total	2022.8	3776 (3644)	0.54 (0.56)	1058.80	1073.14	98.66

Note: a. LG, linkage group, which is numbered in accordance with the corresponding chromosome. b. Number of bins or effective bins (in parenthesis). c. Average distance between adjacent bins or effective bins (in parenthesis). d. Physical span from the first bin to the last bin. e. Full physical length of the chromosome or chromosomal segment. f. Ratio of physical span to full physical length.

**Table 2:** Average ratio of genetic distance to physical distance (cM/Mb) in different regions and the whole span of the genetic map on each eggplant chromosome.

Chromosome	Region I	Region II	Region III	Whole
E01	4.53	0.54	7.31	2.25
E02	-	0.65	4.21	2.15
E03	3.12	0.33	5.38	2.17
E04	5.90	0.32	5.90	2.03
E05	5.60	0.07	5.98	1.53
E06	5.80	0.12	2.84	1.96
E07	10.29	1.27	5.12	2.13
E08	4.13	0.13	3.64	1.60
E09	5.03	0.22	7.31	1.54
E10	5.49	0.19	7.42	1.45
E11	5.89	0.35	7.68	1.69
E12	6.66	0.20	6.52	2.09

Note: For chromosome E04, the average RGPs in region I and region III were estimated based on LG4a+LG4b and LG4b+LG4c, respectively, while that in the whole chromosome was estimated based on LG4a+LG4b+LG4c.

**Table 3:** Mapped QTLs for leaf vein color (LVC) and fruit pericarp color (FPC) in eggplant.

QTL	Season ^a^	Chr.	Pos. (cM)	Bin interval (kb) ^b^	LOD	TLOD ^c^	Add ^d^	Dom ^e^	PVE (%) ^f^
*qLVC4*	1	LG4a	33	B04_76-B04_77 (4070–4120)	4.14	4.03	−0.167	0.000	3.39
*qLVC10*	1	E10	63	B10_240-B10_249	41.06	4.03	−0.710	0.089	56.62
	2	E10	63	B10_240-B10_249	48.27	4.17	−0.746	0.001	72.64
	1+2	E10	63	B10_240-B10_249 (63,480–65,160)			−0.728	0.045	64.63
*qLVC11*	1	E11	2	B11_7-B11_8 (450–660)	4.31	4.03	0.168	0.055	3.46
*qLVC12*	1	E12	42	B12_106-B12_108 (4110–4130)	4.34	4.17	0.026	0.240	3.50
*qFPC6*	1	E06	34	B06_85-B06_88 (2590–2640)	6.87	4.05	−0.376	−0.043	5.97
*qFPC10a*	1	E10	6	B10_18-B10_19 (1190–1220)	4.46	4.05	−0.302	−0.054	3.92
*qFPC10b*	1	E10	63	B10_240-B10_249	37.77	4.05	−1.134	0.085	51.72
	2	E10	63	B10_240-B10_249	38.32	4.05	−1.226	0.049	58.94
	1+2	E10	63	B10_240-B10_249 (63,480–65,160)			−1.180	0.067	55.33

Note: a. 1+2 means the estimates obtained in the two seasons were either averaged or combined. b. The data in the parenthesis indicate the physical interval (in kb) between the start point of the left bin and the end point of the right bin. c. LOD threshold at the overall significance level of 0.05 estimated by permutation tests. d. Additive effect. The negative sign indicates that the allele from the wild parent YQ decreased the trait value. e. Dominance effect. f. Percentage of phenotypic variance explained by the QTL.

## Data Availability

Not applicable.

## Supplementary Materials

The supporting information can be downloaded at: https://www.mdpi.com/article/10.3390/ijms231810258/s1.
