# Supplementary material for: Construction of a High-Density Recombination Bin-Based Genetic Map Facilitates High-Resolution Mapping of a Major QTL Underlying Anthocyanin Pigmentation in Eggplant
Source: Int J Mol Sci. 2022 Sep 6;23(18):10258. doi: 10.3390/ijms231810258 (PMC9499331; doi:10.3390/ijms231810258)
Supplement: Supplementary file 1 [file ijms-23-10258-s001.zip › ijms-1871486-supplementary/Supplemental figure and table/Suppl figures.pdf]

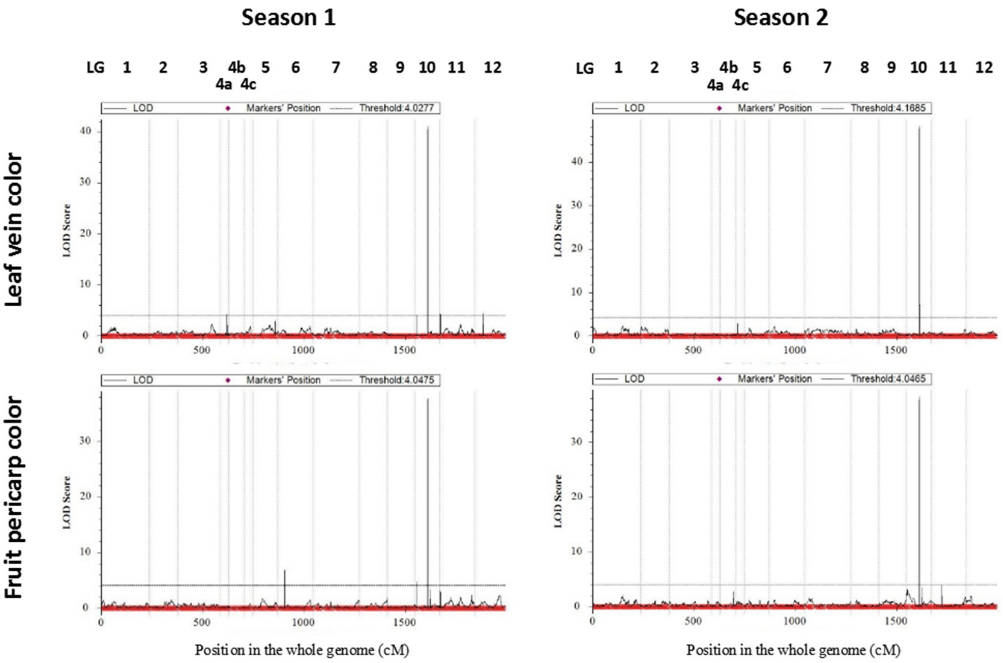

Figure S1 LOD profiles of QTL mapping for two anthocyanin pigmentation traits in eggplant based on the $F_{2:3}$ population grown in two cropping seasons.

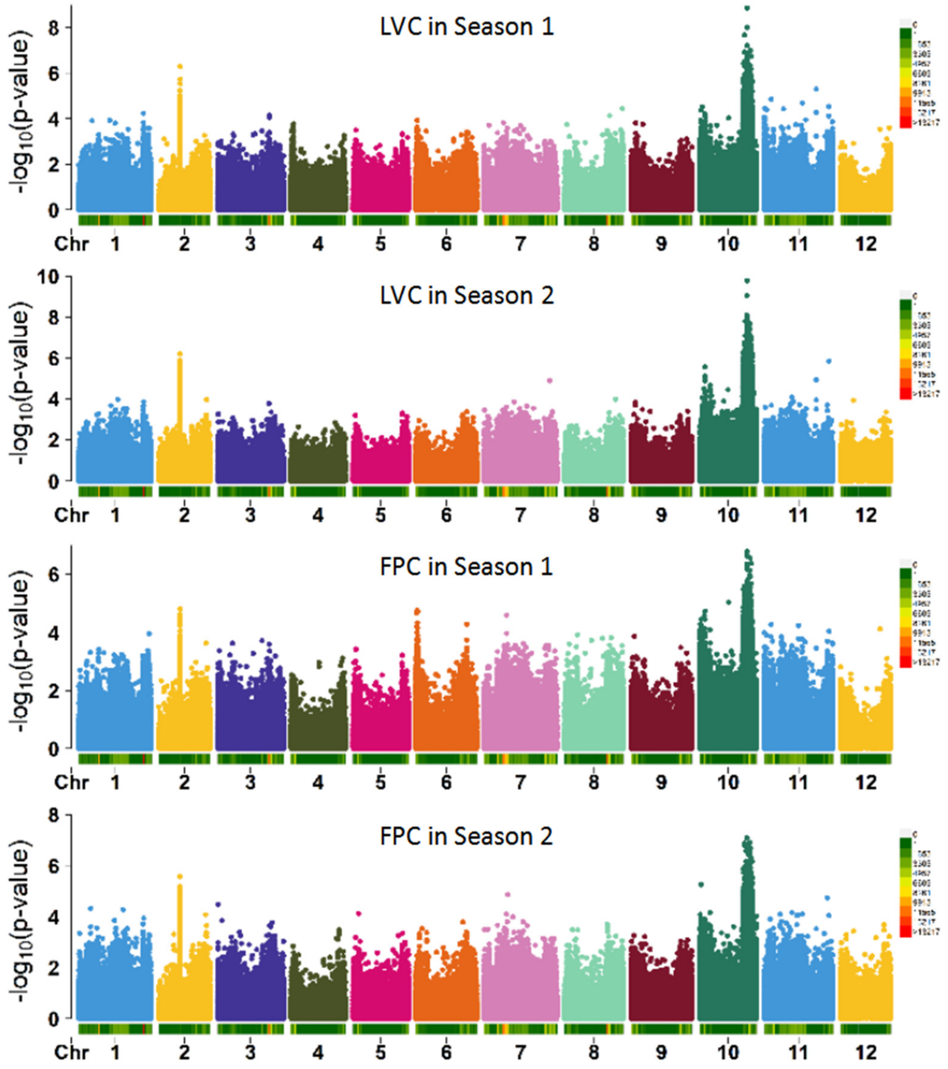

Figure S2 Manhattan plot of genome-wide association analysis for two anthocyanin pigmentation traits in eggplant based on the $F_{2:3}$ population grown in two cropping seasons. The marker density distribution in each chromosome is indicated by a color bar underneath, of which the color scale is on the right.
